# Systemic Lupus Erythematosus is Not Necessarily a Contraindication to Adjuvant Breast Radiation Therapy

**DOI:** 10.7759/cureus.3584

**Published:** 2018-11-13

**Authors:** Kevin Martell, Karen Long, Alexa Solis, Ivo A Olivotto

**Affiliations:** 1 Oncology, University of Calgary, Calgary, CAN; 2 Oncology, Tom Baker Cancer Centre, Calgary, CAN

**Keywords:** breast cancer, lupus, radiotherapy

## Abstract

A 41-year-old woman presented with pT4dN1aM0, right-sided, inflammatory breast cancer. She had a co-morbid diagnosis of systemic lupus erythematosus (SLE) at the age of 20 and was found to have significant kidney involvement (lupus-associated nephritis) at the age of 28. She went on to receive six cycles of neoadjuvant chemotherapy consisting of fluorouracil, epirubicin, cyclophosphamide, and docetaxcel (FEC-D) after which she had radiographically stable disease. She then had definitive treatment with bilateral mastectomy. Pathology showed a 4-cm residual invasive ductal carcinoma in the right breast and three residual metastatic lymph nodes in the right axilla.

After extensive discussions with the patient, which included counseling on the potential increased risk of radiation-induced side effects, she received 50.4 Gy in 28 fractions of adjuvant radiotherapy (RT) to the chest wall and regional lymphatics including the internal mammary chains (IMCs). To minimize the risk of pulmonary toxicity, RT field arrangement consisted of a field-in-field modulated supraclavicular anterior/posterior parallel pair matched to shallow, photon tangent pair with 0.5 cm bolus to the lateral aspect of the chest wall and two matched direct anterior electron fields of 9 MeV with 1 cm bolus and 12 MeV with 0.5 cm bolus medially to cover the remaining residual chest wall and IMCs. This was immediately followed by a boost of 7.5 Gy in three fractions delivered via a photon tangent pair with 1 cm bolus to an area 6 cm superior and inferior to the surgical scar. Total treatment time was 50 days. The patient tolerated the therapy well but she developed grade three acute dermatitis. There were no pulmonary, shoulder joint movement, or brachial plexus side effects.

This case is unusual in that SLE is generally considered a contraindication for elective RT. However, given her high risk for breast cancer recurrence, RT was offered with additional caution to minimize lung dose. Having completed the treatment, the side effects experienced were no greater than what would be expected in someone who did not have a diagnosis of SLE.

## Introduction

Systemic lupus erythematosus (SLE) is a complex autoimmune disease. It can involve several organs including the skin, lungs, pleura, pericardium, joint spaces, renal, hematologic and many other organs/organ systems. Inflammatory reactions within these sites, a propensity for exaggerated immunologic response and poor wound healing can lead many radiation oncologists to withhold RT to the thorax in these patients [1-5].

Literature on the use of adjuvant chest wall or regional nodal RT for breast cancer in patients with SLE is limited to case reports from the two dimensional (2D) planning era [1-3, 6]. We present the case of a patient treated in 2017 where underlying tissue was maximally spared.

## Case presentation

**Figure 1:**
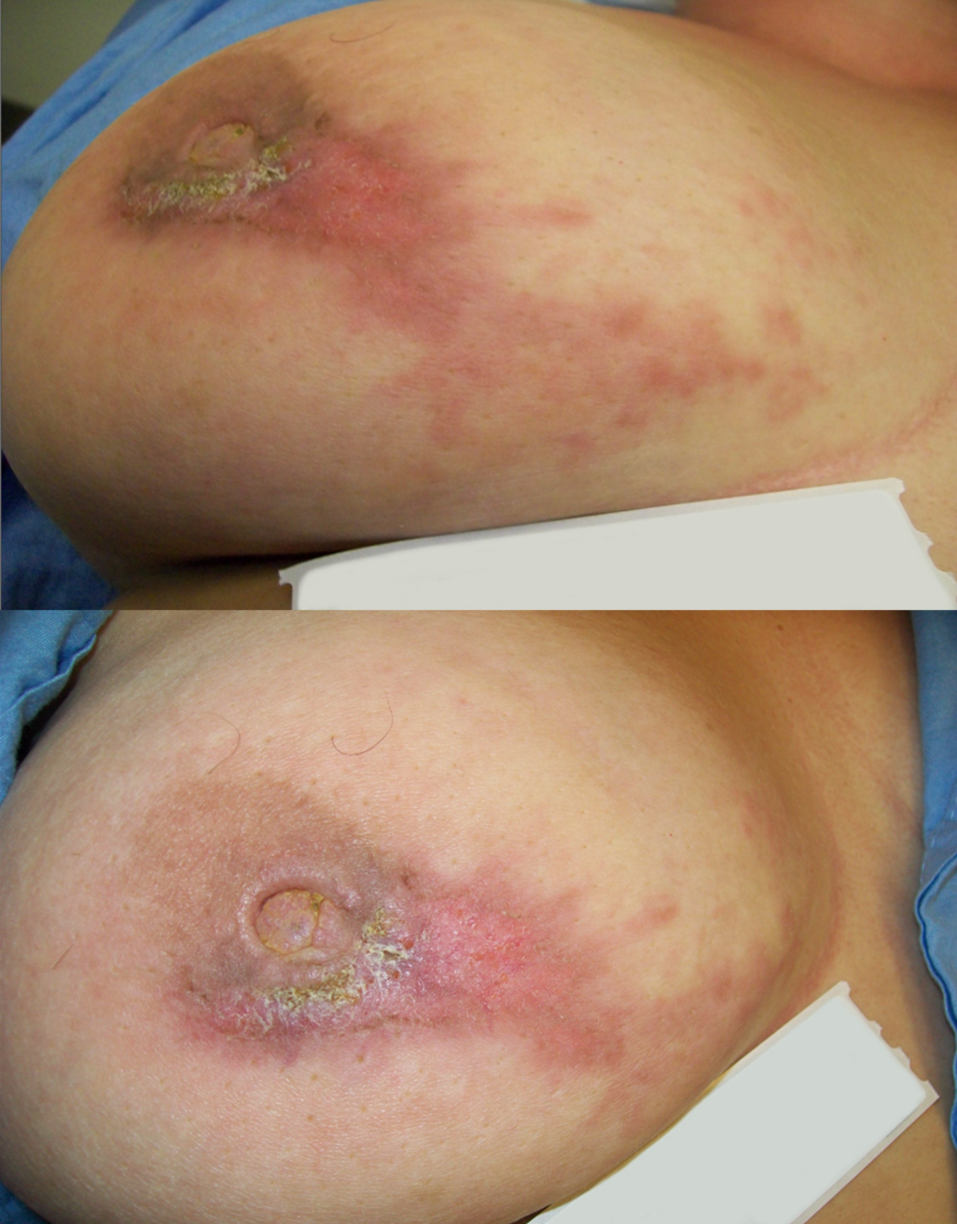
Preoperative medial (top) and anterior (bottom) view of right breast in a 41-year-old patient with controlled systemic lupus erythematosus and breast cancer.

A 41-year-old female presented with a two-month history of right breast erythema and nipple erosion (Figure 1). Needle core biopsy showed a grade two invasive ductal carcinoma; estrogen receptor 8/8, progesterone receptor 4/8, and human epidermal growth factor receptor two negative via silver in situ hybridization. There was extensive lymphovascular and dermal invasion. Staging workup with axillary ultrasound, chest and abdomen computed tomography (CT), and bone scan revealed a conglomerated lymph node mass measuring 1.5 cm in the right level I-II axilla but no distant metastases (cT4dN1M0 [IIIC]). Thirteen years earlier she had presented with Raynaud’s phenomena, arthralgias, alopecia, malar rash, lupus nephritis, and thrombotic thrombocytopenic purpura. She was diagnosed with SLE according to the American College of Rheumatology criteria and was treated with plasmapheresis, six months of cyclophosphamide, and 24 months of mycophenolate mofetil. She was then placed on irbesartan and maintenance hydroxychloroquine. Over subsequent years her SLE had remained stable with no other organ involvement. A summary of her autoimmune disease activity is listed in Table 1.

**Table 1:** Lupus disease involvement and markers of activity at time of diagnosis or throughout the course of SLE and last known status at time of treatment with external beam radiotherapy. *microACR: micro albumin creatinine ratio, ratio was performed in 2007 as at diagnosis with lupus, the patient was in complete renal failure from nephritis requiring dialysis. **Complete blood count results were interpreted from dictation at the time of systemic lupus erythematosus diagnosis. Exact values are not available in the current health records system.

Marker/Criterion	Diagnosis	Radiotherapy	Reference range
Malar rash	Yes	No	
Discoid rash	No	No	
Photosensitivity	Yes	Yes	
Oral ulcers	No	No	
Nonerosive arthritis	Yes	No	
Pleuritis or pericarditis	--	--	
Pleuritis	No	No	
Pericarditis	No	No	
Renal disorder	--	--	
Proteinuria (g/day or ACR)	28.0 (microACR)*	0.0	microACR: 0.0-3.4
Cellular casts	Yes	No	
Neurologic disorder	--	--	
Seizures	No	No	
Psychosis	No	No	
Hematologic disorder			
Hemoglobin (g/L)	Normal**	146	120-160
White blood cells (10E9 N/mL)	Normal**	4.2	4-11
Lymphocytes (10E9 N/mL)	Normal**	0.7	0.5-3.3
Thrombocytes (10E9 N/mL)	<100**	228	150-400
Immunologic disorder			
Anti-DNA/anti dsDNA	Positive	1 (dsDNA)	0-9
Anti-smooth muscle	Negative	Negative	
Antiphospholipid antibodies (either serum IgG or IgM anticardiolipin antibody levels; lupus anticoagulant or treponema pallidium immobilization false positive)	Positive	Negative	
Antinuclear antibody	1:320 (positive)	1:80 (negative)	Negative
C-reactive protein level	--	--	--
Eosinophil sedimentation rate	--	--	--
Extractable nuclear antigen (ENE)	Positive ribosomal-P + centromere-P	Negative	Negative
Compliment component 3 (C3) (g/L)	1.05	1.36	0.60-1.60
Compliment component 4 (C4) (g/L)	0.34	0.40	0.10-0.40

The patient received three cycles of three weekly fluorouracil, epirubicin, and cyclophosphamide followed by three cycles of three weekly docetaxel with clinically stable disease. One month later she had a right total mastectomy, sentinel node biopsy with completion right-sided level I-II axillary dissection, and a prophylactic left-sided mastectomy. Pathology showed a 4-cm residual tumor and 3/15 nodes positive for residual disease (ypT4dN1aM0R0).

The patient healed well after surgery and after thorough discussion which included the indications and risks of adjuvant RT in the setting of well-established SLE, the patient elected to proceed with RT.

**Figure 2:**
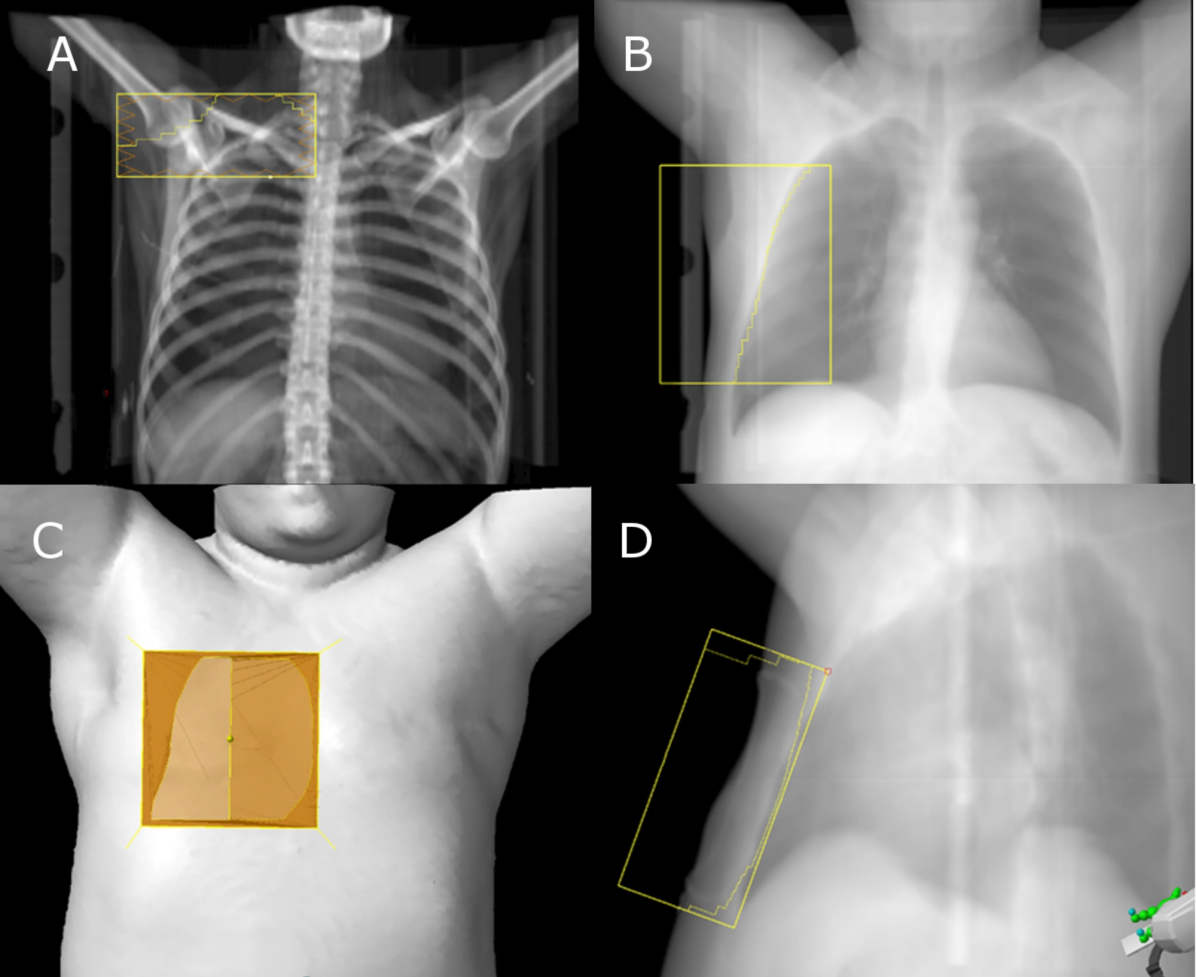
Beams eye view of fields for supraclavicular anterior beam (A), wide lateral shallow primary anterior beam (B), 12 MeV electron block (C) (medial), 9 MeV electron block (C) (lateral) and narrow (boost) lateral tangent beam (D). 9 MeV electron beam was matched to the lateral aspect of the wide tangent beam.

Setup and treatment fields for RT were designed to minimize lung and cardiac doses. All nonboost RT treatments were performed with deep inspiration breath hold technique and used a combination of electron and photon fields (Figure 2). The plan was field based on 3D CT simulation image sets and aimed to cover a clinical target volume including the chest wall and regional lymphatics. No planning target volume was defined but was accounted for with direct placement of fields. Organs at risk were contoured and included: thyroid, heart, ipsilateral lung, contralateral lung, and liver. The axillary level II-III and supraclavicular nodal regions were treated with an anterior-posterior, parallel opposed pair of fields using field-in-field planning and a mixture of 6 and 15 MV energies to improve homogeneity. A single isocenter was placed at the inferior margin of the nodal fields and was matched to a mixed 6 and 15 MV photon tangent pair to treat the lateral chest wall and level I-II of the axilla. This was matched and feathered medially to an anterior 9 MeV electron beam. A second, 12 MeV electron beam was matched to the medial aspect of the 9 MeV beam to provide internal mammary coverage. A 0.5 cm bolus was used for the lateral chest wall photon beams and the 12 MeV electron beam and 1 cm bolus were used for the 9 MeV electron beam. A 12 cm, clinically delineated boost volume around the surgical scar was treated with a shallow, 6 MV, tangent pair and 0.5 cm bolus using free breathing.

**Figure 3:**
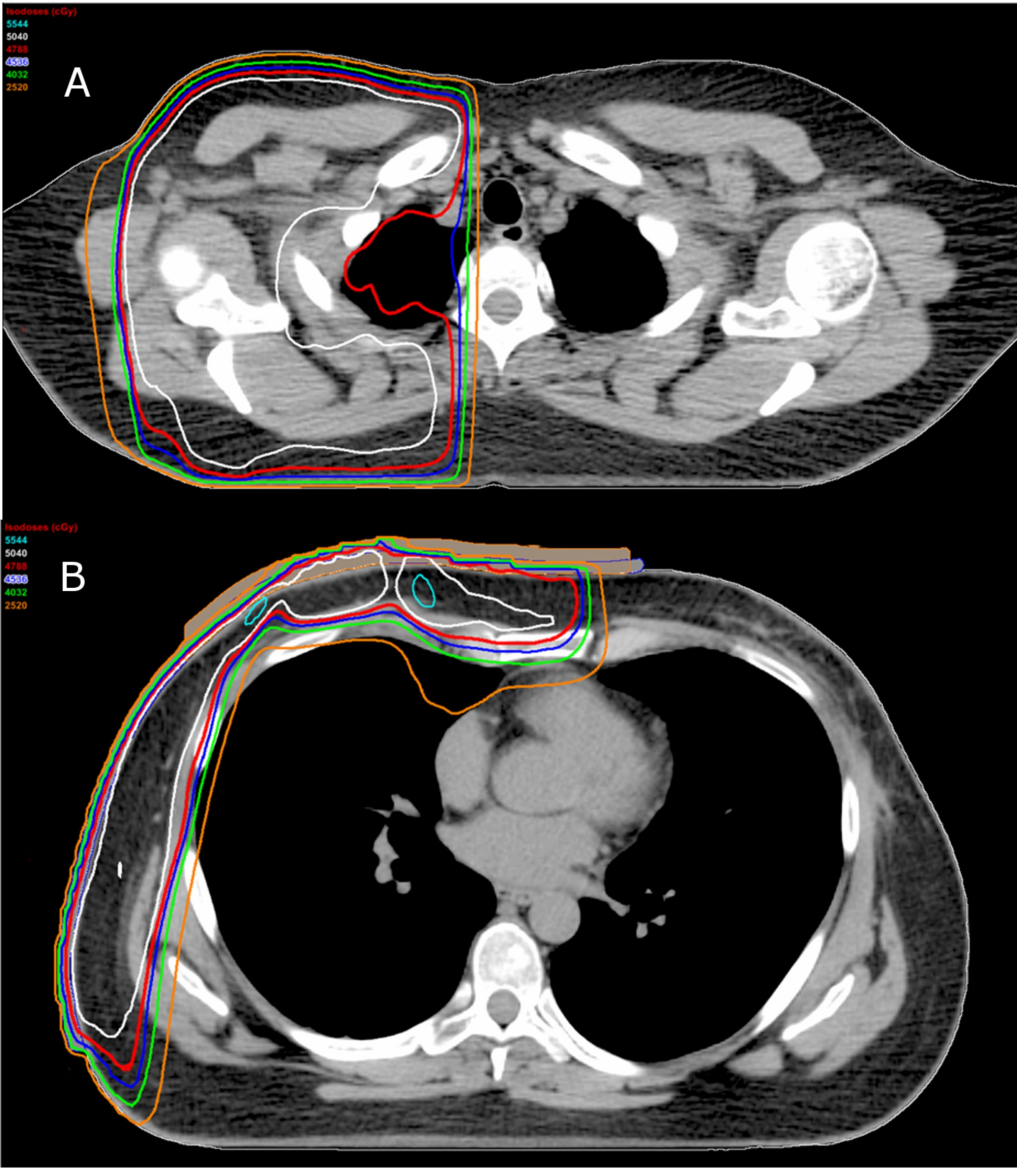
Representative dose distributions at level of supraclavicular parallel opposed pair (A) and lateral tangent chest wall pair (B). Cyan: 5544cGy (110%) | White: 5040cGy (100%) | Red: 4788cGy (95%) | Blue: 4536cGy (90%) | Green: 4032cGy (80%) | Orange:  2520cGy (50%)

The final isodose distribution for the primary treatment (excluding boost) is shown in Figure 3. Prescribed dose was 50.4 Gy in 28 fractions (50.4/28) followed by 7.5/3 to the boost volume. The right lung volume receiving 20Gy (V20) and V5 were 13% and 65% (local clinical constraints are V20<30% and V5<80% for the ipsilateral lung). Mean heart dose was 3.59 Gy (clinical constraint: mean dose<4.0 Gy). Thyroid was blocked from the primary fields and received only scatter dose. Varian Medical Systems’ Eclipse software™ version 13.6 (Varian Medical Systems Inc, Palo Alto, USA) was used. Electron and photon dosimetry were calculated using Monte Carlo and the Anisotropic Analytical Algorithm, respectively.

**Figure 4:**
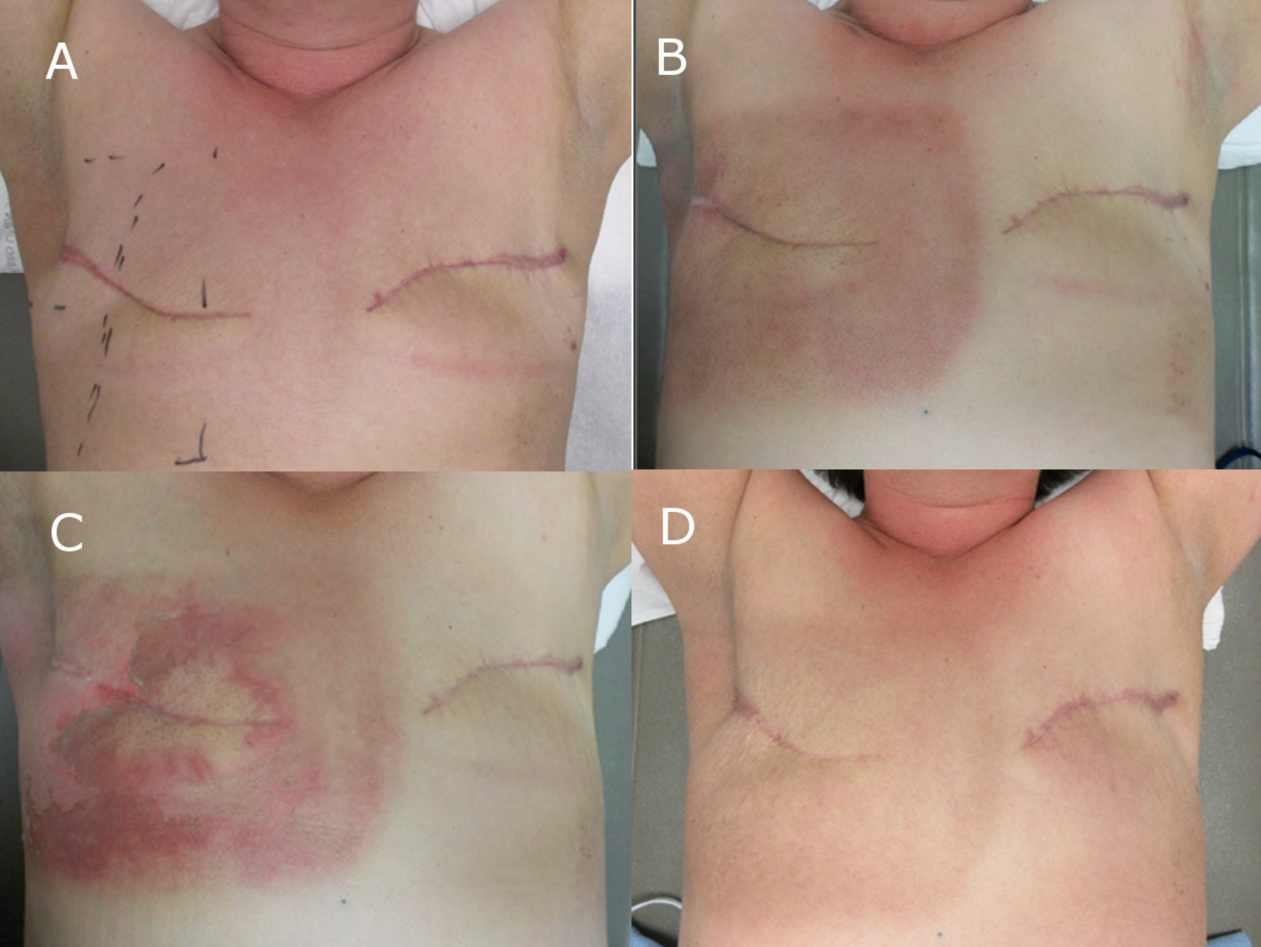
Dermatitis reaction at (A) start of treatment (photon field position marked), (B) completion of therapy, (C) one week post completion of therapy and (D) 12 months post completion of therapy. The patient did not develop any pneumonitis symptoms following radiotherapy.

The patient completed RT over 50 elapsed days then started adjuvant exemestane with three-monthly leuprolide injections. She developed CTCAE v4 grade one and subsequently, grade three dermatitis by days 20 and 57 [7]. This was managed conservatively with twice daily Glaxal Base (R) dressings and daily aeration of the breast. It resolved, leaving her with anhidrosis and mild tanning (Figure 4). CT imaging showed only subtle lung scarring and she did not develop clinical evidence of pneumonitis, pericarditis, right upper extremity lymphedema, symptoms of brachial plexopathy or any other signs or symptoms attributable to RT toxicity. At one year she was free of local and systemic recurrence.

## Discussion

This 41-year-old woman with well-documented but stable SLE and locally advanced inflammatory breast cancer received adjuvant RT to her chest wall and regional lymphatics without undue or unexpected side effects. Sophisticated treatment planning techniques were used to minimize normal tissues receiving high-dose RT while still delivering a therapeutic dose to the target volume.

The literature regarding adjuvant breast RT in patients with SLE is sparse. Benk et al. reported on two such patients receiving RT following breast conservation [2]. One patient received 50/25 with a 10/5 electron boost and the other received 40/16 followed by a 12.5/5 electron boost. With 12 months of follow-up, no acute or late side effects were reported.

Ross et al. reported on a patient with SLE treated for breast cancer who later died of congestive heart failure secondary to pericarditis [8]. In another report Robertson et al. treated with 52.5/26 followed by a 46 hour, 16 Gy boost delivered via Ir-192 implant. The patient required mastectomy for severe pain, swelling, erythema, and breast contracture [6]. 

Fleck et al. treated two patients with Cobalt-60 to the chest wall and regional lymphatics [3]. The first who received 40/20 before treatment was terminated for severe moist desquamation. The patient had limited arm range of motion long-term. In the second, 45/22 resulted in severe skin ulceration then bronchopleural-cutaneous fistula and osteoradionecrosis of the clavicle six years after treatment.

These patients were all treated with 2D planning and in no patient was the autoimmune rheumatologic disease activity well described. In the current report, the patient had been reviewed by her rheumatologist and nephrologist, both of whom reported good disease control and had no objections to the oncologic treatments for her breast cancer. This is important because, as shown by Gold et al. risks of chronic RT toxicity seem to be dependent on autoimmune rheumatologic disease activity defined by the number of organs involved [9]. The current patient would be considered as having low severity disease at the time of treatment and tolerated RT well.

## Conclusions

The study reports on a woman with concurrent diagnoses of controlled SLE and high-risk breast cancer. She was treated with neoadjuvant systemic therapy, a modified radical mastectomy with axillary dissection and standard-dose adjuvant RT to the chest wall and regional nodes without unexpected toxicities. The benefits of RT may outweigh the risks of toxicity in well-selected patients with SLE.

## References

[1] Giaj-Levra N, Sciascia S, Fiorentino A (2016). Radiotherapy in patients with connective tissue diseases. Lancet Oncol.

[2] Benk V, Al-Herz A, Gladman D, Urowitz M, Fortin PR (2005). Role of radiation therapy in patients with a diagnosis of both systemic lupus erythematosus and cancer. Arthritis Rheum.

[3] Fleck R, McNeese MD, Ellerbroek NA, Hunter TA, Holmes FA (1989). Consequences of breast irradiation in patients with pre-existing collagen vascular diseases. Int J Radiat Oncol Biol Phys.

[4] Olivotto IA, Fairey RN, Gillies JH, Stein H (1989). Fatal outcome of pelvic radiotherapy for carcinoma of the cervix in a patient with systemic lupus erythematosis. Clin Radiol.

[5] Hölscher T, Bentzen SM, Baumann M (2006). Influence of connective tissue diseases on the expression of radiation side effects: a systematic review. Radiother Oncol.

[6] Robertson JM, Clarke DH, Pevzner MM, Matter RC (1991). Breast conservation therapy. Severe breast fibrosis after radiation therapy in patients with collagen vascular disease. Cancer.

[7] (2018). Common Terminology Criteria for Adverse Events (CTCAE) Version 4.03. https://evs.nci.nih.gov/ftp1/CTCAE/CTCAE_4.03.

[8] Ross JG, Hussey DH, Mayr NA, Davis CS (1993). Acute and late reactions to radiation therapy in patients with collagen vascular diseases. Cancer.

[9] Gold DG, Miller RC, Pinn ME, Osborn TG, Peterson IA, Brown PD (2008). Chronic toxicity risk after radiotherapy for patients with systemic sclerosis (systemic scleroderma) or systemic lupus erythematosus: association with connective tissue disorder severity. Radiother Oncol.

